# Prevalence of asymptomatic *P. falciparum *gametocyte **carriage among school children in Mbita, Western Kenya and assessment of the association between gametocyte density, multiplicity of infection and mosquito infection prevalence.

**DOI:** 10.12688/wellcomeopenres.16299.2

**Published:** 2021-04-13

**Authors:** Abdoulie O. Touray, Victor A. Mobegi, Fred Wamunyokoli, Hellen Butungi, Jeremy K. Herren

**Affiliations:** 1 Department of Molecular Biology and Biotechnology, Institute of Basic Sciences, Technology and Innovation, Pan African University (PAUSTI), Nairobi, Kenya; 2 International Centre of Insect Physiology and Ecology (icipe), Nairobi, Kenya; 3 Department of Biochemistry, School of Medicine, University of Nairobi, Nairobi, Kenya; 4 Department of Biochemistry, Jomo Kenyatta University of Agriculture and Technology (JKUAT), Nairobi, Kenya; 5 Wits Research Institute for Malaria, School of Pathology, Faculty of Health Sciences, University of the Witswatersrand, Johannesburg, South Africa

**Keywords:** P. falciparum, asymptomatic, gametocyte density, MOI, mosquito infection prevalence, Mbita

## Abstract

**Background:** **Asymptomatic *Plasmodium falciparum *gametocyte carriers are reservoirs for sustaining transmission in malaria endemic regions. Gametocyte presence in the host peripheral blood is a predictor of capacity to transmit malaria. However, it does not always directly translate to mosquito infectivity. Factors that affect mosquito infectivity include, gametocyte sex-ratio and density, multiplicity of infection (MOI), and host and vector anti-parasite immunity. We assess the prevalence of gametocyte carriage and some of its associated risk factors among asymptomatic schoolchildren in Western Kenya and to further analyse the association between gametocyte density, multiplicity of infection (MOI) and mosquito infection prevalence.

**Methods:** *P. falciparum *parasite infections were detected by RDT (Rapid Diagnostic Test) and microscopy among schoolchildren (5-15 years old). Blood from 37 microscopy positive gametocyte carriers offered to laboratory reared *An. gambiae s.l.* mosquitoes. A total of 3395 fully fed mosquitoes were screened for *Plasmodium* sporozoites by ELISA. *P. falciparum *was **genotyped using 10 polymorphic microsatellite markers. The association between MOI and gametocyte density and mosquito infection prevalence was investigated.

**Results:** **A significantly higher prevalence of *P. falciparum *infection was found in males 31.54% (764/2422) ( *p*-value < 0.001) compared to females 26.72% (657/2459). The microscopic gametocyte prevalence among the study population was 2% (84/4881). Children aged 5-9 years have a higher prevalence of gametocyte carriage (odds ratios = 2.1 [95% CI = 1.3–3.4], *P* = 0.002) as compared to children aged 10-15 years. After offering gametocyte positive blood to *An. gambiae s.l.* by membrane feeding assay, our results indicated that 68.1% of the variation in mosquito infection prevalence was accounted for by gametocyte density and MOI (R-SQR. = 0.681, *p* < 0.001).

**Conclusions:** **We observed a higher risk of gametocyte carriage among the younger children (5-9 years). Gametocyte density and MOI significantly predicted mosquito infection prevalence.

## Introduction

The intensification of global and local malaria control measures has led to marked reduction in disease burden in many regions including sub-Saharan Africa. The incidence of *Plasmodium falciparum* clinical cases and prevalence have declined by 40% and 50%, respectively, within the African continent between 2000 and 2015 ^1^. However, recent data indicates this trend might be reversing, with an estimated 213 million malaria cases and 380,700 related deaths in the World Health Organisation (WHO) African Region between 2017 and 2018, an increase relative to previous years ^2^. Clearly, malaria continues to be a very serious public health problem on the African continent, threatening the lives of many people, particularly children and pregnant women. In Kenya, like many other African countries, *P. falciparum* is the dominant parasite species with about 70.2% of the population at risk of the disease ^3^. Malaria is one of the leading causes of hospital admissions and death in the country, accounting for about 30% and 19% outpatient and inpatient cases, respectively, with an estimated inpatient death of 3–5% ^2, 4^.

The Kenyan government, through the implementation of a national strategic malaria control plan, and subsequently by the launching of the next iteration of its national malaria strategy (KMS) 2019—2023, has intensified its fight against the disease in a bid to attain a **“**malaria free Kenya **”**. This involved the introduction and scaling up of interventions such as long-lasting insecticide nets (LLINs), rapid diagnostic tests (RDTs), and artemisinin-based combination therapy (ACT) ^5, 6^. The implementation of these interventions has resulted in a decline in malaria transmission in many parts of the country ^7^. Nevertheless, the coastal part of the country and areas along the shores of Lake Victoria continue to face very high malaria transmission ^8^.

Malaria parasite transmission from humans to the mosquito vectors requires the presence of infectious mature gametocytes in the peripheral blood of the human host ^9, 10^. Based on the central role of gametocytes in propagating and sustaining malaria transmission, the prevalence of gametocytes and their densities are often used as surrogate indicators for the disease transmission potential ^11, 12^. The advent of highly sensitive molecular tools has enabled us to understand that every malaria positive individual is a current or prospective gametocyte producer and therefore, has some transmission potential. Studies in malaria endemic and high transmission areas have reported higher asexual parasite and gametocyte prevalence and densities in children relative to adults ^13, 14^. In high malaria transmission settings, due to repeated parasite exposure, older children and adults develop immunity against the parasite ^15, 16^. As a result, these age groups are most likely to experience asymptomatic infections harboring gametocytes at microscopic and sub-microscopic densities, thereby serving as efficient parasite reservoirs for sustaining malaria transmission ^12, 14, 17^. Reports about high prevalence of asymptomatic infections and gametocyte densities in schoolchildren have been documented in some malaria endemic areas ^17, 18^. Asymptomatic malaria infections in schoolchildren mostly remain undiagnosed and are not treated due to the lack of clinical manifestation. Therefore, this group of people are largely neglected by most of the currently implemented malaria interventions and control programs ^17, 18^. In addition, following the decline in malaria burden in many endemic areas, information on the prevalence of asymptomatic *P. falciparum* infections and gametocyte carriage in schoolchildren, particularly in remote settings in sub-Saharan Africa, remains patchy ^19^. Since asymptomatic infections and prevalence of gametocyte carriage in schoolchildren may significantly hamper the attainment of malaria control and elimination goals in sub-Saharan Africa ^18, 20^, it will be important to further investigate dynamics and infectivity of asymptomatic carriers.

The presence of gametocytes in the peripheral blood of the human host does not necessarily translate into mosquito infectivity ^9, 21^. Some of the major factors that influence the successful transmission of *P. falciparum* gametocytes to the mosquito vectors include, human attractiveness and exposure to the mosquito vectors, host and vector immune responses, seasonality, gametocyte maturity and densities, and multiplicity of infection (MOI) ^21, 22^. MOI is the number of distinct parasite clones concurrently infecting a host. The link between MOI and gametocytemia of *P. falciparum* is still not fully elucidated ^21^; however, some studies have reported a positive association between MOI and gametocyte carriage ^14, 23^. The presence of multiple genetically diverse *P. falciparum* clones is reported to increase the chances of some parasite clones to evade the host anti-parasite immune responses, thereby promoting gametocyte development and persistence ^14, 24^. Studies have reported that mosquito infectivity is positively correlated to gametocyte density and primarily determined by female gametocyte density, however, transmission at low gametocyte densities can be limited by density of male gametocytes ^25– 28^. However, the proportion of variation in mosquito infection prevalence that can be explained by gametocyte density and MOI has not been fully elucidated.

 In malaria endemic settings, asymptomatic infections characterized by high rates of polyclonal infections and variations in gametocyte carriage among different age categories is not uncommon ^15, 29, 30^. Variations in gametocyte densities among the different age categories can be partly explained by the age-related anti-parasite immunity due to repeated exposure among the elderly children and adults age groups21. In order to accelerate malaria elimination , interventions geared towards interrupting the parasite transmission through efficient and effective identification and treatment of both asymptomatic and symptomatic parasite carriers will be of immense importance ^14, 15^. Understanding the association between gametocyte density, MOI and mosquito infectivity will enhance proper identification of parasite reservoirs responsible for sustaining the ongoing malaria transmission in the region ^14^. Here, we report on the prevalence of gametocyte carriage and some of its associated risk factors among asymptomatic schoolchildren (age 5–15 years) in western Kenya and further assesses the association between gametocyte density, MOI and mosquito infection prevalence.

## Methods

### Ethics and consent

Parents or guardians of the children signed an informed consent form for participation in the study, having data analysed and publication of results. In addition, assent was obtained from older children between the ages of 12 and 15. The Kenya Medical Research Institute (KEMRI) Scientific and Ethics Review Unit (SERU) granted approval for the original study (KEMRI/RES/7/3/1). All experiments were performed in accordance with the relevant guidelines and regulations.

### Study site

**Figure 1.**
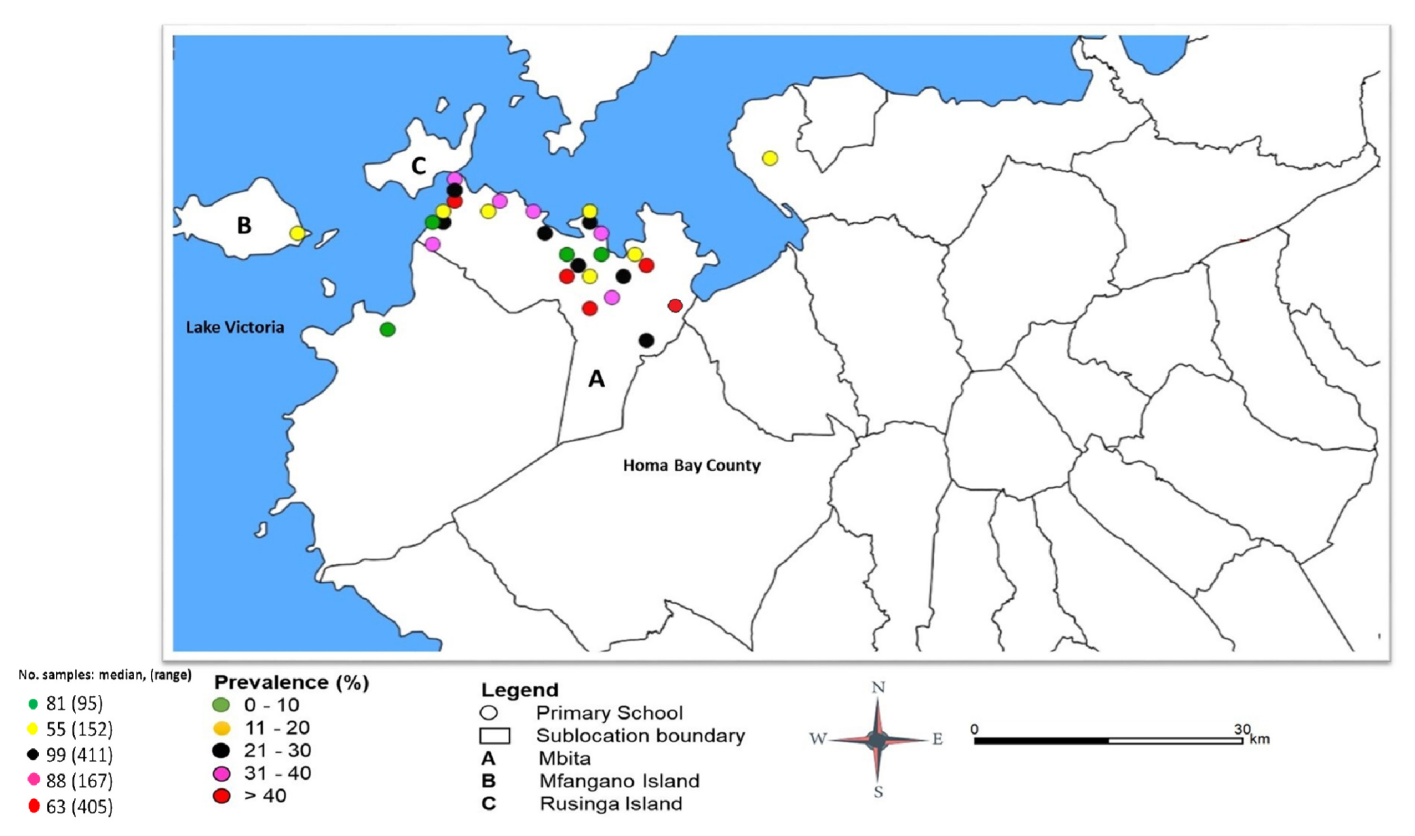
Map of Homa Bay County indicating the prevalence of *Plasmodium falciparum* infection among the schools in the study site. The site-specific prevalence (%) was calculated as the percentage of *P. falciparum* positive infections within each school.

This cross sectional study was carried out in the Homa Bay County of Western Kenya formerly, the Nyanza province. Study participants were recruited from primary schools (41 primary schools) primarily within Mbita sub-county (within 50 km radius of Mbita town). The sub-county is situated on the shores of Lake Victoria and located between latitudes 0° 21’ and 0° 32’ South and longitudes 34° 04’ and 34° 24’ East. The area of the district is about 163.28 km ^2^ with a population of 124,938 ( Figure 1). The compound is the main residential unit and mostly comprises of one or more households. The houses are a mix of traditional mud grass thatch huts and modern concrete and corrugated iron houses. Fishing, farming, and animal rearing are the major economic activities in the region. Perennial malaria transmission is reported in the region. The peak transmission occurs in July and relatively lower transmission levels are reported from November to January ^31^.

### Study subjects and sample collection

Primary schoolchildren between the ages five and 15 years residing in Homa bay county, Western Kenya were recruited and screened for *P. falciparum* malaria infection using a rapid diagnostic test (RDT) (SD Bioline Malaria Ag P.f/Pan HRP-II/pLDH) (Standard Diagnostics Ref 05FK60, Inc; Suwon City, Republic of Korea) and microscopy. Schoolchildren from the various primary schools in Mbita subcounty and the neighbouring villages (within 50 KM) were enrolled in a study that commenced in December 2016 to evaluate the effects of symbiotic microbes and mosquito vector competence. The samples analysed in this study were collected from December 2016 to December 2018. The inclusion criteria used for the sampling included being at primary school in Mbita or any of the surrounding villages within 50 Km of Mbita between the ages of 5–15 years and not showing any of the symptoms of malaria during screening. 

Blood samples were collected by a clinician from each participant in their various schools for RDT and 10% Giemsa stained thin and thick blood films preparation for microscopy diagnosis of *P. falciparum* malaria infection. Microscopy was carried out *in-situ* and all the stained slides were then well packaged and transported to *icipe* TOC Mbita campus for storage. Gametocytes were counted against 1000 white blood cells and the counts converted to parasites/μL assuming a density of 8000 WBCs/μL. In addition, 100 μL of blood was collected on filter papers (two spots per paper) (Whatman 3MM; Whatman, Maidstone, United Kingdom) for DNA extraction. The filter paper dried blood spots (DBS) were stored at -20°C. Participants who were found by microscopy to carry *P. falciparum* gametocytes were contacted by the clinician through their parents/guardians for further sample collection on the same day. An additional 4 mL of venous blood was collected from participants by the clinician for use in the membrane feeding assays. A total of 4881 participants were screened in this study. This sample size was obtained based on the number of study participants within the designated study area that consented to partake in the study over the study period. The samples included in this study were originally collected to investigate differences in levels of infectivity between laboratory colony mosquitoes and the progeny of wild caught mosquitoes, genetic diversity, and multiplicity of infection ^32^, this study is a sub-study that was concurrently undertaken using the same samples.

### Experimental infection of mosquitoes

A modified version of the standard membrane feeding assay protocol published elsewhere was used in this study ^9^. In brief, larvae (G _1_) from *icipe-*TOC insectary colonies of *An. gambiae s.s.* (G _0_) were reared at 30.5°C (+/- 2°C) and 30% humidity at the insectary of *icipe* TOC Mbita campus. Venous blood samples (4 mL) collected in heparin tubes from each *P. falciparum* gametocyte positive individual were immediately fed to the mosquitoes. Experimental feeds were carried out in batches of 100 female *An. gambiae s.s.* mosquitoes (3–5 day-old) per feeding cup.The mosquitoes were fed on a total volume of 500 *µ*l of blood via an artificial membrane attached to a 14 mm water-jacketed glass feeder (Chemglass, USA) maintained at 37°C. All the membrane feeding assays were carried out at the *icipe* Mbita TOC campus. A total of 37 gametocyte-positive venous blood samples collected from different individuals (only those that consented to provide the extra 4 mL of blood) were used to feed the mosquitoes. After 15–20 minutes, fully fed mosquitoes are selected and kept on glucose for seven days at 27°C – 29°C. On the tenth day post-infection, the mosquitoes that were alive from each feeding experiment were then collected and stored at -20°C in Eppendorf tubes and were later individually tested for *P. falciparum* carriage using ELISA. The proportion of infected mosquitoes was determined by detecting the *P. falciparum* circumsporozoite protein (CSP) in the stored mosquito samples using a modified CSP ELISA protocol adapted from ^33^. In brief, mosquitoes stored in 1.5 mL Eppendorf tubes were incubated in 50 μL grinding buffer followed by homogenization in the buffer solution by thorough grinding using a pestle. The homogenized samples were transferred to plates (Corning Cat. No. 2797) and stored at -20°C overnight. Each of the ELISA plates was coated with 50 μL MAb capture antibodies (0.5 mg/mL Capture Monoclonal Ab Pf2A10-CDC, CAT #: MRA-890, MR4/ATCC, Virginia, USA). Dilution specifications for *P. falciparum* used here is the same as previously published and were incubated overnight at room temperature ^33, 34^. 200 μL of the blocking buffer was added to each well after removing the Mab then incubated for one hour at room temperature 50 µL of the mosquito homogenates was added to each well . The positive controls (Pf-PC, BEI resources, Virginia, USA). were serially diluted using blocking buffer because they are free of *Plasmodium* parasites. Negative controls were insectary-reared male mosquitoes ground up in blocking buffer. A two-hour incubation of the plates was carried out at room temperature after which, the plates were washed four times with 200 μL PBS-Tween using ELx50 ELISA washer (BioTek Instruments, Winooski, Vermont, U.S.A). ABTS Substrate Component containing solutions A and B were mixed in a 1:1 ratio to a final volume of 20 ml/plate (SeraCare 5120-0032). The monoclonal antibody (MAb) peroxidase conjugate 27 (0.5 mg/mL Peroxidase Labelled Mouse Monoclonal Ab Pf2A10-CDC, CAT #: MRA-890, MR4/ATCC, Virginia, USA) was prepared in specified concentrations for *Plasmodium falciparum* at a volume of 40 μL in 10 mL blocking buffer for each plate. 100 μL of the MAb peroxidase conjugate was added to each well after drying the plates, wrapped in an aluminium foil then incubated in a dark place at room temperature. After which the plates were washed four times using PBS-Tween, dried and 50 μL substrate solution added to each well followed by 30 minutes incubation at room temperature The plates wereread on ELx808 ELISA reader (BioTek Instruments, Winooski, Vermont, U.S.A) using Gen 5 3.0 Software (BioTek Instruments, Winooski, Vermont, U.S.A) at a wavelength of 405 nm to determine optical density values of the samples. An optical density (OD) cut-off values for CSP positivity were computed by the addition of three standard deviations to the mean OD value of the CSP-negative distribution from each plate ^34^. OD values of each plate were adjusted by pooling the negative controls together, then the pooled negative mean OD value determined and subtraction of this pooled mean OD value from the mean negative OD value per plate to obtain the specific correction value per plate. The unique correction value was then added/subtracted from all OD readings in each respective plate to normalize readings across plates. Standard curves of absorbance against sporozoite concentration were generated for each plate using the serial diluted positive controls. Quantification of samples was computed using the equation generated from the standard curve and their corresponding absorbance values.

### Microsatellite genotyping

Genomic DNA (gDNA) was extracted from the DBS samples using the QIAamp DNA Mini Kit (Cat # 51304, QIAGEN, Hilden, Germany) based on the manufacturer’s protocol. gDNA quality and concentration of each sample was determined using a Nanodrop 2000C (Thermo Fisher Scientific, Waltham, MA, USA) and samples were stored at -20°C until used. The microsatellite amplification, fragment analysis and MOI determination method is based on a previous study ^35^. In brief, genomic DNA (gDNA) was extracted from filter paper dried blood spots samples using QIAamp DNA Mini Kit (CAT #: 51304, QIAGEN, Hilden, Germany). gDNA samples were genotyped using primer sets (See Table S1, *Extended data* ^36^) targeting 10 polymorphic microsatellite markers via a hemi nested PCR protocol using 5X FIREPol Master Mix (Solis BioDyne, Estonia) in a SimpliAmp Thermal Cycler (Applied Biosystems, Loughborough, UK). A total reaction volume of 20 µL was prepared for the hemi one PCR and the components are as follows; 1X FIREPol Master Mix (CAT #: 04-11-00115, Solis BioDyne, Estonia), 0.3 µM forward primer, 0.3 µM reverse primer (Macrogen, South Korea) and 10 ng/µL of the template DNA. The hemi one PCR conditions include a 2 min initial denaturation at 94°C; 30 cycles of 30 sec at 94°C, 30 sec at 42°C, 30 sec at 40°C and 30 sec at 65°C; then a 5 min final elongation at 65°C. The hemi two reaction was also run in a 20 µL total reaction volume containing 1X FIREPol Master Mix (CAT #: 04-11-00115, Solis BioDyne, Estonia), 0.4 µM of each primer and 5 µL of hemi one amplicons and the reaction condition includes; 2 min initial denaturation at 94°C; 30 cycles of 30 sec at 94°C, 30 sec at 45°C and 30 sec at 65°C and 5 min final elongation at 65°C. ABI 3730XL (Applied Biosystems) was used for the separation of hemi 2 PCR products using GeneScan 400HD ROX Size Standard (Applied Biosystems, Foster City, CA). GeneMarker V3.0.1 software (SoftGenetics, LLC) was used for scoring and quantification of allele sizes and peak heights, respectively ^37^. The samples analysed here are part of those used in our previous study ^32^. These are filter paper dried blood spots collected from the study participants as described above. A total of 37 samples (samples used for the membrane feeding assays) were genotyped for this analysis. 

### Data storage and analysis

Age, gender, weight and *Plasmodium* parasitemia of each study participant together with mosquito infection prevalence and microsatellite genotyping data were obtained. Descriptive statistics and Pearson Chi-Square test for significance between groups were determined. Risk factors analysis was done using a binary logistic regression model and multiple correlation and regression analysis was used to determine the regression coefficients, statistical significance of regression model ( *t* value), and proportion of mosquito infection prevalence (dependent) contributed by independent variables (gametocyte density and MOI) derived from the multiple coefficient of determination (R ^2^). The mosquito infection prevalence was determined as the percentage of mosquitoes infected with *P. falciparum* parasite after successfully feeding on the naturally infected human blood. Statistical analyses were conducted in IBM SPSS Statistics for Windows, version 25 (IBM Corp., Armonk, N.Y., USA). Schools were mapped using geographical information system (GIS) and the map generated using QGIS software version 2.4.0. Rainfall data for Mbita (0° 25' 0" South, 34° 12' 0" East) were obtained from Climate Engine, Desert Research Institute and University of Idaho, accessed on 08/04/2020 ^38^.

## Results

### Demographic and parasitological characteristics of the study participants

In this study, a total of 4881 schoolchildren (age 5–15 years) were screened using RDT and the parasite status confirmed by microscopy. The total number of female and male participants were 2459 and 2422, respectively. Regarding the parasitological characteristics of the study participants, significant differences were observed among males and females, with higher *P. falciparum* prevalence among the males [male: 53.76% (764/1421); female: 46.24% (657/1421); *p*-value < 0.001]. However, considering the age versus sex distribution of gametocyte carriage, no significant difference was found by comparing the male (5-9 years) [53.65% (382/712)] to female (5–9 years) [46.35% (330/712)] and male (10-15 years) [53.88% (382/709)] to female (10-15 years) [46.12% (327/709)] ( *p*-value = 0.958). There was also no statistically significant difference in *P. falciparum* parasite carriage between the age groups [5–9 yrs.: 50.10% (712/1421); 10–15 yrs.: 49.89% (709/1421); *p*-value = 0.072] ( Table 1). The total number of mixed infections ( *P. falciparum* plus *P. ovale* and/or *P. malariae*) detected in the study population was 204, with a non-significant difference between the age groups [5–9 years: 15.73% (112/712); 10–15 years: 12.98% (92/709); *p*-value = 0.139], while there were 1217 single infections ( *P. falciparum* only). Most of the mixed infections were found in females compared to males [females: 16.74% (110/204); males: 12.30% (94/764); *p*-value = 0.017] ( Table 2).

**Table 1.** Parasitological characteristics of the study participants.

Variables	Age group (years)		Gender	
	5 – 9	10 – 15	Female	Male
Positive	50.10% (712/1421)	49.89% (709/1421)	46.24% (657/1421)	53.76% (764/1421)
Negative	52.98% (1833/3460)	47.02% (1627/3460)	52.08% (1802/3460)	47.92% (1658/3460)
**χ2 (p-value)**	3.328 (0.072)		13.770 (< 0.001) *	

Percentage of *P. falciparum* positive and negative infections among the study participants. χ ^2^ = Pearson's chi-squared test and (*) indicates statistical significance.

**Table 2.** Characteristics of the positive infections.

Variables	Age group (years)		Gender	
	5 – 9	10 – 15	Female	Male
Mixed infection	15.73% (112/712)	12.98% (92/709)	16.74% (110/657)	12.30% (94/764)
Single infection ( *P.falciparum* only)	84.27% (600/712)	87.02% (617/709)	83.26% (547/657)	87.70% (670/764)
**χ2 (p-value)**	2.192 (0.139)		5.661 (0.017) *	
Asexual	48.99% (655/1337)	51.01% (682/1337)	46.75% (625/1337)	53.25% (712/1337)
Gametocyte	32.14% (27/84)	3.81% (27/709)	38.10% (32/84)	61.90% (52/84)
**χ2 (p-value)**	11.253 (0.001) *		2.380 (0.123)	
**Population gametocyte prevalence** 2% (84/4881)				
**Gametocyte prevalence (** ***P. falciparum*** **positives) ** 6% (84/1421)				

Population gametocyte prevalence is the percentage of gametocyte carriers among the total study population ( *P. falciparum* positive and negative samples together), while the gametocyte prevalence among the *P. falciparum* positive samples is the percentage of gametocyte carriers among the *P. falciparum* positive samples only (excluding *P. falciparum* negatives). χ ^2 ^= Pearson’s chi-squared test and (*) indicates statistical significance.

**Figure 2.**
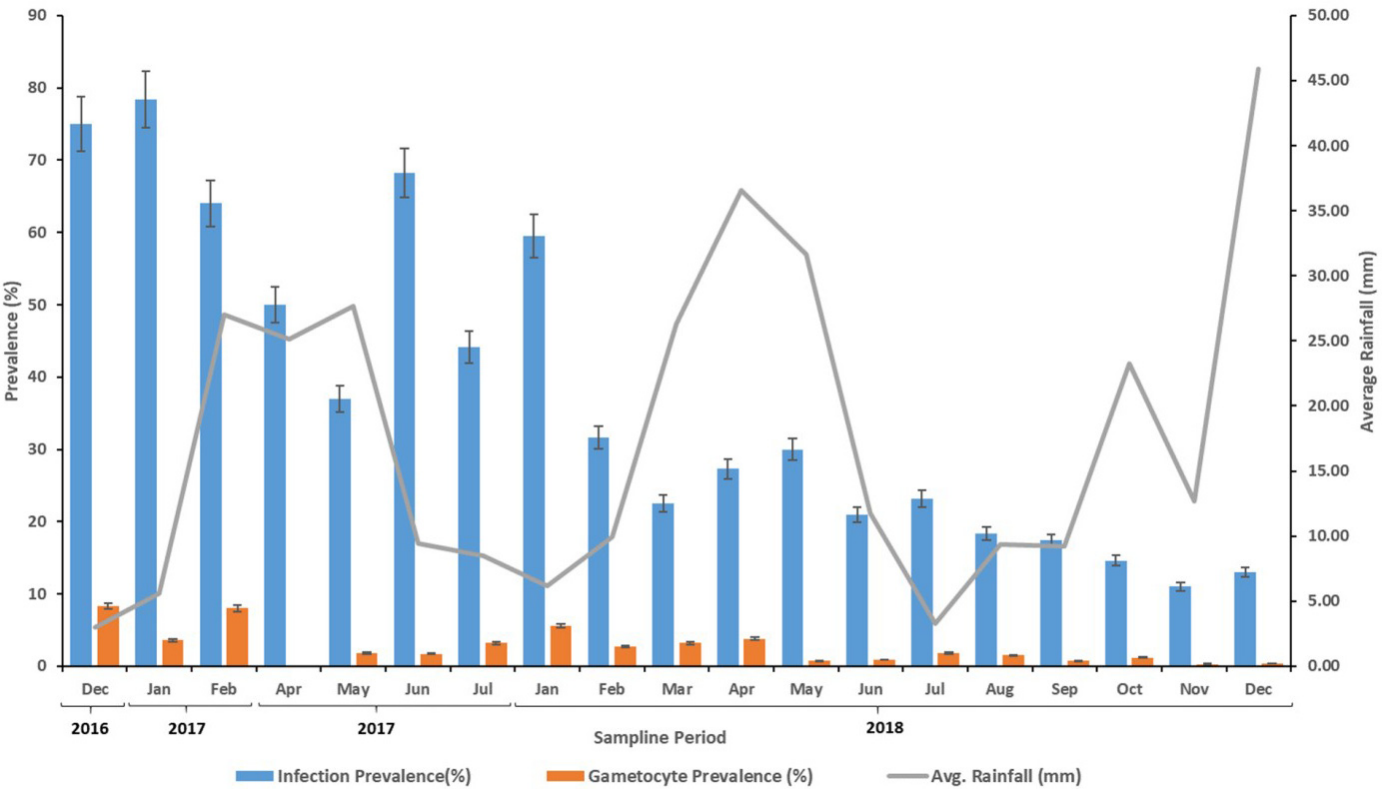
*P. falciparum infection* (blue) and gametocyte (brown) prevalence among the study participants and average rainfall (gray) during the various sampling periods.

The population *P. falciparum* prevalence in this study calculated as the percentage of *P. falciparum* infections within the study sample was 29.11% (1421/4881). The level of *P. falciparum* carriage varies among study sites (range: 0–100%, *p*-value < 0.001) and across sampling periods (range: 11–78.4%, *p*-value < 0.001, Figure 1 and Figure 2).

### Microscopy gametocyte p and associated risk factors in the study population

The total number of gametocyte carriers as detected using microscopy in the study was 84/4881, with 57 of the carriers found within the age group 5–9 years as compared to 27 in the age-group 10–15 years ( *p*-value = 0.001, Table 2). The microscopy gametocyte prevalence among the *P. falciparum* malaria carriers (only *P. falciparum* positive individuals) was found to be 6% (84/1421). These represent the minimum gametocyte prevalence levels, due to the sensitivity limits of microscopy. The gametocyte carriage in females (38.10%, 32/84) and males (61.90%, 52/84) was not significantly different ( *p*-value = 0.123). The *P. falciparum* infection rate and gametocyte positive rate both follow a gradual declining trend from 2016 to late 2018. However, a high *P. falciparum* infection rate does not always coincide with a high gametocyte positive rate, for example samples from June 2017 and April 2018. In addition, the *P. falciparum* infection rate does not appear to be heavily influenced by rainfall ( Figure 2).

The analysis showed that risk of *P. falciparum* infection was highest among the males as compared to females [OR = 0.8 (95% CI = 0.7–0.9), *P* < 0.001] while the age of an individual was not an independent risk factor. However, children between the ages of 5–9 years have a higher risk of gametocyte carriage when infected with *P. falciparum* as compared to those between the ages 10–15 years [OR = 2.1 (95% CI = 1.3–3.4), *P* = 0.002].

### Relationship between gametocyte density and multiplicity of *Plasmodium falciparum* infections (MOI) and mosquito infection prevalence

**Figure 3.**
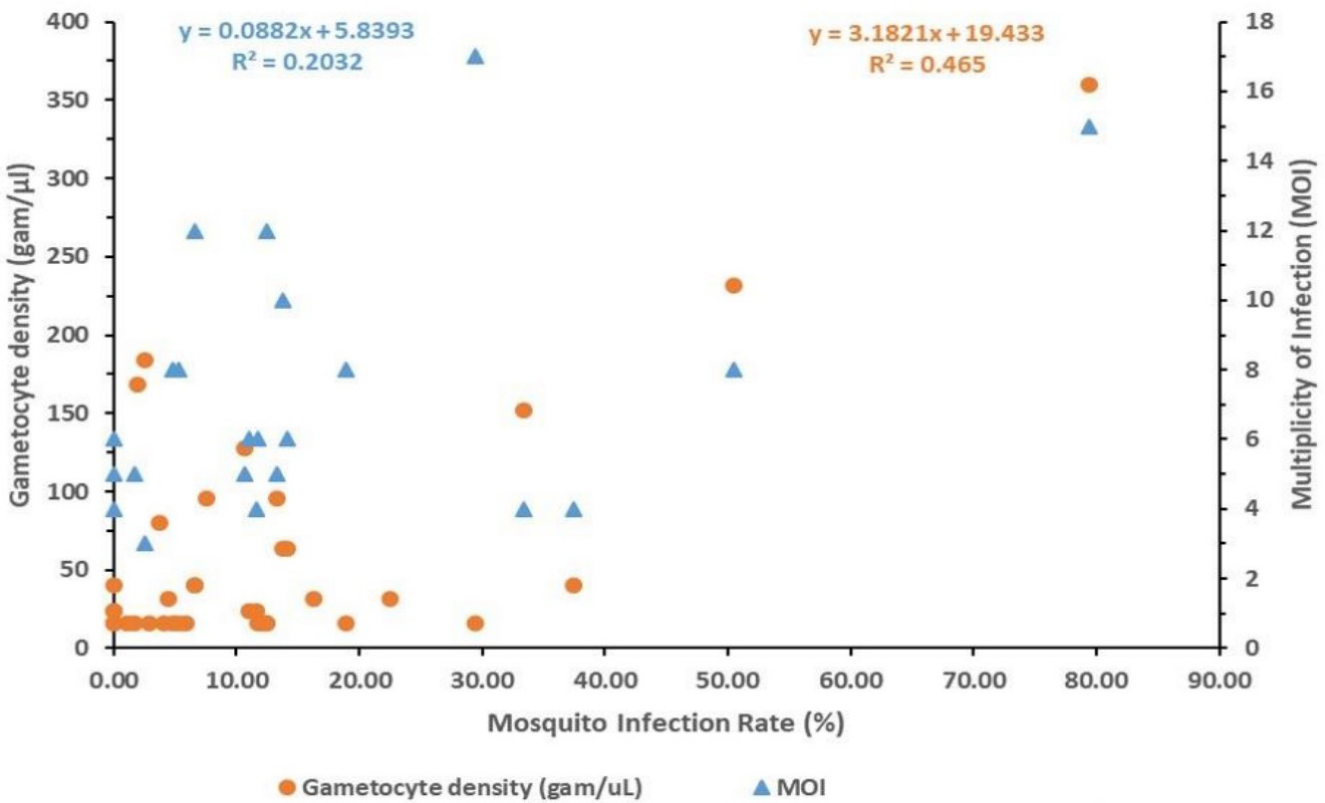
Relationship between gametocyte density (gametocyte/µL) and multiplicity of infection (MOI) with mosquito infection rate.

The total number of samples used in assessing the relationship between gametocyte density, MOI and mosquito infection prevalence was 37. However, 15 of the 37 samples failed to amplify during the microsatellite amplification PCR and are recorded as missing data. After the feeding experiments, 3395 mosquitoes were used in the CSP ELISA assay. All the blood samples offered to the mosquitoes have resulted in at least one infection. 463 mosquitoes were infected recording a mean infection rate of 12.71% (Median: 7.6, IQR: 10.97, SE: 2.63, SD: 16.1) and mean gametocyte density was 59.89 gametocytes µL -1 (Median: 24, IQR: 48, SE: 12.28, SD: 74.71), while the mean number of distinct alleles per isolate was 7.32 (Median: 6, IQR: 3, SE: 0.80, SD: 3.76) (see density and MOI data, *Underlying data* ^35^). In this study, a significant positive correlation was found between *P. falciparum* gametocyte densities in the patient blood samples and mosquito infection prevalence (0.682, *p*-value < 0.0001). In addition, a positive correlation between multiplicity of *P. falciparum* infection (MOI) and mosquito infection prevalence was reported (0.451, *p*-value = 0.035). Notably, the correlation between MOI and gametocyte density was not statistically significant (0.167, *p*-value = 0.459). The mosquito infection prevalence is defined as the percentage of infected mosquitoes after day 10 of the membrane-feeding assay ( Table 3 and Figure 3).

**Table 3.** Multiple correlation analysis of gametocyte density and multiplicity of *P. falciparum* infection (MOI) with the infection prevalence in the mosquitoes.

Parameters	Infection rate ( *P*-value)	Gametocyte density ( *P*-value)	MOI ( *P*-value)
Infection prevalence	1	0.682 (< 0.0001) *	0.451 (0.035) *
Gametocyte density	0.682 (< 0.0001) *	1	0.167 (0.459)
MOI	0.451 (0.035) *	0.167 (0.459)	1

The dependent variable in this analysis is the infection prevalence. *Ref* represents the reference, (*) denotes statistical significance. Gametocyte density (Gam/μL) is presented without adjustment and the MOI is presented as a continuous variable.

A multiple regression was run to predict mosquito infection prevalence from gametocyte density (gametocyte/µL) and MOI ( Table 4). These variables statistically significantly predicted mosquito infection prevalence, *F*(2, 19) = 20.235, *p* < 0.0001, *R ^2^* = 0.681 and both contributed significantly to the prediction, *p* < 0.05.

**Table 4.** Parameter of multiple linear regressions analysis.

Parameters	Coefficients	Std. Error	t-statistic	*P*-value
(Constant)	-6.644	5.564	-1.194	**0.247**
Gametocyte density (gam/μL)	0.151	0.028	5.328	**< 0.001**
MOI	1.707	0.672	2.54	**0.020**

***R = 0.825, R-SQR. = 0.681, Adj. R-SQR = 0.647, SE = 11.418***. ***R*** is the multiple correlation coefficient, ***R-SQR.*** (R-square) is the multiple coefficient of determination, ***Adj. R-SQR*** represents the adjusted R-square, and ***SE*** is the standard error. MOI, multiplicity of infection. Gametocyte density (Gam/ μL) is presented without adjustment and the MOI is presented as a continuous variable.

The multiple coefficient of determination (R-SQR. = 0.681) indicated that about 68.1% of the variation in mosquito infection prevalence is accounted for by the gametocyte density and MOI. Thus, the formulated equation for mosquito infection prevalence in this study is:

$$\hat{\text{Y}}\;=\;\text{-6.644}\;+\;\text{0}{\text{.151X}}_{\text{1}}\;+\;\text{1}{\text{.707}\;\text{X}}_{\text{2}}$$

Where $\hat{\text{Y}}$ is the expected mosquito infection prevalence, and X _1_ and X _2_ are the gametocyte density and MOI, respectively.

## Discussion

We monitored the prevalence of gametocyte carriers and investigated risk factors among asymptomatic schoolchildren (age 5–15 years) in Western Kenya. An assessment of the relationship between gametocyte density, MOI and mosquito infection prevalence was also carried out. We found a moderate and declining rate of gametocyte prevalence in the study population, which is in agreement with the findings of other studies in the region ^39, 40^. Intensification of the fight against malaria in the region by the Kenyan government may be contributing to the decline in positivity rate and gametocyte carriage reported in our study ^41^. Gametocyte prevalence was higher among the younger age groups (5–9 years), which accounted for 67.86% (57/84) of the total gametocyte carriers in the study population. Similar patterns of gametocyte carriage was reported by other studies ^21, 31^. This could be due to age-dependent development of anti-parasite immunity due to repeated exposure in endemic settings ^21, 31^. The high prevalence of gametocyte carriage among the younger age group (5–9 years) pinpoints the potential role of this age group in sustaining malaria transmission in the region. Children have been reported to be important contributors to the malaria infectious reservoir in many other settings ^21^. Among the *P. falciparum* gametocyte positive individuals, males tended to be slightly overrepresented 61.9% (52/84) as compared to females 38.1% (32/84)]. However, this is not statistically significant. The *P. falciparum* prevalence was much lower in 2018 when compared to the 2017 season. This is likely due to an indoor residual spraying (IRS) campaign conducted by Africa Indoor Residual Spraying (AIRS) Kenya, in early 2018 in this region ^42^. Nonetheless, gametocyte prevalence remained at moderate levels during all the sampling periods, indicating year-round gametocyte carriage in the study population irrespective of the rainfall levels and pattern. In malaria endemic settings, asymptomatic carriers are known to harbour gametocytes even during the non-transmission season and are reported to be responsible for the resurgence of malaria infections during the subsequent transmission season ^38^. When combined with prevalent *Anopheles* mosquito vectors, asymptomatic *P. falciparum* gametocyte carriage can lead to perennial transmission of malaria in the region.

The only independent risk factor associated with *P. falciparum* infection found in this study was gender. Males have higher odds of *P. falciparum* infection in the study area as compared to females. Gender was reported as a risk factor in other studies in the region ^31^. This finding is in line with the reports that female children are biologically less susceptible to infectious diseases as compared to male children ^43^. Age was not found to be a risk factor for contracting *P. falciparum* malaria infection in this study but was linked with gametocyte carriage when infected with *P. falciparum.* Younger children (5–9 years) have a higher risk of gametocyte carriage when infected with *P. falciparum*. A study in Tanzania has also reported similar a association of age with increased gametocyte prevalence ^44^.

A significant positive association was found between gametocyte density and mosquito infection prevalence (correlation coefficient = 0.682, *p*-value < 0.001). Infection prevalence tends to be relatively higher among mosquitoes that fed on carriers with high gametocyte densities (> 20 Gam/uL). This result corroborates the findings of other studies ^21, 45^. In particular, it has been noted that over relatively low gametocyte densities, in the range observed in this study, an increase in gametocytaemia corresponds with a rapid increase in the proportion of infected mosquitoes ^26, 28^. This maybe a specific parasite strategy to maximize human-mosquito transmission (fertility assurance) ^46, 47^.

The relationship between multiplicity of *P. falciparum* infection and mosquito infection prevalence is not well documented. We found that *P. falciparum* isolates harbouring multiple distinct clones positively influence the mosquito infection prevalence, since there was a significant positive correlation between MOI and mosquito infection prevalence (correlation coefficient = 0.451, *p*-value = 0.035). In contrast, a negative association between MOI and mosquito infection prevalence and intensity has been reported elsewhere ^48^. In our study, the interaction between MOI and gametocyte density was not statistically significant, which is in line with other studies ^48^.

Although gametocyte density is clearly an important factor in predicting the success of *P. falciparum* transmission to the mosquito vector, gametocyte density alone in blood samples does not equate to their infectiousness to mosquitoes ^49^. Therefore, understanding the association between gametocyte density and other parasite parameters like MOI with mosquito infection prevalence will improve our understanding of the dynamics of *P. falciparum* transmission. Our results indicate a significant and positive combined effect of the explanatory variables (gametocyte density and multiplicity of *P. falciparum* infection) on the mosquito infection prevalence [ *F*(2, 19) = 20.235, *p* < 0.0001, *R ^2^* = 0.681]. These results show that MOI and gametocyte density account for about 68.1% of the variation in mosquito infection prevalence. This may be linked to intense inter-strain competition due to increased investment in gametocytes and multiple clone infections (MOI) that may trigger the emergence of highly transmissible or virulent parasite strains thereby increase mosquito that may trigger the emergence of highly transmissible or virulent parasite strains thereby increase mosquito infectivity ^50– 52^. Another plausible explanation for the association between MOI, gametocyte density and mosquito infection prevalence found in this study may be due to the outcome of strategic balancing between in-host survival and between-host transmission ^47, 53– 55^. At relatively low MOIs, the level of intra-host competition is relatively low and the *P. falciparum* parasites reduce conversion rates to enhance asexual replication and in-host survival through reproductive restraint. However, at high MOIs, the intra-host competition is too intense for reproductive restraint and the parasites tend to increase the conversion rate to facilitate between-host transmission ^53, 54^. The high mosquito infection prevalence observed at high MOIs can be explain by the maximised gametocyte production to increase the chances of between-host transmission.

## Limitations of the study

The major limitation of the study is the small sample size used in determining the association between gametocyte density, MOI and mosquito infection prevalence. This was partly due to the limited number of study participants who consented to providing extra venous blood samples for the mosquito feeding assays. The asexual parasite density was not determined due to the study design centred on gametocyte density and MOI. The study used microscopy in determining gametocyte presence and density. However, microscopy is known to be of relatively low resolution, hence some gametocyte carriers might not be detected ^56^. The membrane feeding assay might have low mosquito feeding efficiency when compared to direct skin feeding, however, ethical approval for this study required the use of membrane feeding assays ^21^.

## Conclusions

Malaria prevalence and gametocyte carriage is high among asymptomatic schoolchildren, particularly the younger age group (5–9 years), in the region. The relatively stable and year-round prevalence of gametocyte carriage among the study participants in this study signals the role of schoolchildren in maintaining malaria transmission in the study area. The statistically significant and positive combined effect of the explanatory variables on the mosquito infection prevalence will help in determining the human infectious reservoirs in different malaria endemic settings. Malaria control interventions that are highly efficient in reducing multiple clone parasite carriage and gametocyte density could aid in disrupting the transmission of the parasite, thereby facilitating the ultimate elimination of the disease in the region. 

## Data availability

### Underlying data

Figshare: Data supporting a study of the prevalence of asymptomatic *P. falciparum* gametocyte carriage in schoolchildren and assessment of the association between gametocyte density, multiplicity of infection and mosquito infection prevalence. https://doi.org/10.6084/m9.figshare.13048088 ^36^.

This project contains the following underlying data:

- Study participants screening data_1.xlsx (NB: Single = *P. falciparum* only, Mixed = *P. falciparum* plus either *P. ovale* or *P. malariae* or both)
- Gam density_MOI_Infection prevalence_3.xlsx.
- Sampling period_Infection and Gam prevalence_2.xlsx.
- Raw ELISA output data.xlsx
- Raw ELISA output data_OD values.xlsx
- Raw genotyping output data.xlsx

### Extended data

Figshare: Data supporting a study of the prevalence of asymptomatic *P. falciparum* gametocyte carriage in schoolchildren and assessment of the association between gametocyte density, multiplicity of infection and mosquito infection prevalence. https://doi.org/10.6084/m9.figshare.13048088 ^36^.

This project contains the following extended data:

- Supplementary file Table S1.docx.
- Sampling site and period analysed data.docx

Data are available under the terms of the Creative Commons Zero “No rights reserved” data waiver (CC0 1.0 Public domain dedication).
