# Supplementary material for: Prevalence of asymptomatic P. falciparum gametocyte carriage among school children in Mbita, Western Kenya and assessment of the association between gametocyte density, multiplicity of infection and mosquito infection prevalence
Source: Wellcome Open Res. 2021 Apr 13;5:259. Originally published 2020 Oct 29. [Version 2] doi: 10.12688/wellcomeopenres.16299.2 (PMC8078214; doi:10.12688/wellcomeopenres.16299.2)
Supplement: Peer review history

# Peer review: Reviewer response for version 2

**DOI:** 10.21956/wellcomeopenres.18379.r43474

**Published:** 2021-04-27

**Authors:** Lisa Ranford Cartwright

**Affiliations:** 1 Institute of Biodiversity, Animal Health and Comparative Medicine, College of Medical, Veterinary and Life Sciences, University of Glasgow, Glasgow, UK

The authors have not responded to my review, and the revised manuscript does not address the main issues I raised. I cannot, therefore, change my original view. For example, the errors in the MOI table have not been explained or corrected (and the analysis then redone if necessary with the correct MOI numbers), and the additional data on mosquito numbers dissected have not been provided.

 I also note that the revised Table 1 now does not make sense. For example, in the table, the number of 5-9 year olds who are parasite positive is given as 712/1421, but those who are negative is now 1833/3460. Why is the denominator different?

 Also in the text, there is probably a new mistake in the text/table 1 between gametocyte positive and *P. falciparum* positive (unless all P.f positive are also gametocyte positive) - for example, the text says that the *P. falciparum* prevalence among the males is 764/1421, but the gametocyte carriage is given as 382/712 in the 5-9 year olds and 382/709 in the 10-15 year olds ie total 764 gametocyte positives, so all Pf positive are gametocyte positive?

 These errors need to be corrected before indexing.

Is the work clearly and accurately presented and does it cite the current literature?

Partly

If applicable, is the statistical analysis and its interpretation appropriate?

Partly

Are all the source data underlying the results available to ensure full reproducibility?

Yes

Is the study design appropriate and is the work technically sound?

Partly

Are the conclusions drawn adequately supported by the results?

Partly

Are sufficient details of methods and analysis provided to allow replication by others?

No

Reviewer Expertise:

Genetics and transmission biology of P. falciparum.

I confirm that I have read this submission and believe that I have an appropriate level of expertise to state that I do not consider it to be of an acceptable scientific standard, for reasons outlined above.

# Peer review: Reviewer response for version 2

**DOI:** 10.21956/wellcomeopenres.18379.r43473

**Published:** 2021-04-26

**Authors:** Teun Bousema

**Affiliations:** 1 Department of Medical Microbiology, Radboud University Nijmegen Medical Center, Nijmegen, The Netherlands

The revised manuscript has addressed many of my comments. I have a few outstanding comments.

 I still find figure 1 difficult to grasp. The prevalence estimates are difficult to interpret without exact confidence intervals or number of observations. The legend now gives prevalence and range but it would be more informative to let the size of the dot be informed by the number of observations, or some other way of presenting uncertainty in estimates.

 The ELISA protocol is now better explained. I would still really like to see how convincing the positive mosquitoes were. Ten days after feeding is appropriate, by then positive mosquitoes should be highly positive. It is important to present (either in main text or supplemental info) the set threshold for positivity and the OD range of mosquitoes that are concluded to be positive. This is important to interpret indirect assessments of infection status.

 Table 1 should be simplified. Male and females are mutually exclusive categories here. So percentage female is enough.

 Table 2 should also be clarified. I am not sure what the X2 value relates to since it is presented under asexual parasites and gametocytes. The denominators also appear mixed. So each colum should have (roughly) the same denominator that is the percentage of individuals in a certain categorie (e.g. with a single clone infection (not single infection, which is a strange term) expressed as a percentage of the age group. Population gametocyte prevalence and gametocyte prevalence among Pf positives should not be in the table but in the main text.

 Comparisons between age groups (e.g. ‘The total number of gametocyte carriers as detected using microscopy in the study was 84/4881, with 57 of the carriers found within the age group 5–9 years as compared to 27 in the age-group 10–15’) are sometimes difficult to follow. This specific example suggests a count analysis while I suspect prevalences are compared. It should be followed by a more in-depth analysis of gametocyte prevalence for a given parasite density. The currently phrasing suggests an effect of age on gametocyte production but this may of course be fully driven by age-dependent differences in parasite density.

 The statement ‘However, a high *P. falciparum* infection rate does not always coincide with a high gametocyte positive rate, for example samples from June 2017 and April 2018. In addition, the *P. falciparum* infection rate does not appear to be heavily influenced by rainfall‘, requires formal analyses.

 MOI and gametocyte density need to be incorporated in a multi-variate model. The current phrasing is not sufficient. Gametocyte density is positively associated, MOI is positively associated. The two are not statistically significantly associated but there is still a need for an appropriate multivariate model to interpret the findings.

 Table 3 can be omitted.

 Table 4 can be omitted.

 The formula is probably too simplistic to do justice to the non-linear association between variables. The appropriate model is more complex than currently described (see Bradley *et al.*) and has DIC as indicator of fit to determine whether adding MOI will, for instance, improve model fit compared to gametocyte density alone. With uncertainties around gametocyte density and MOI and the limited number of observations, I feel the 68% is too precise and can be omitted.

Is the work clearly and accurately presented and does it cite the current literature?

Yes

If applicable, is the statistical analysis and its interpretation appropriate?

Partly

Are all the source data underlying the results available to ensure full reproducibility?

Yes

Is the study design appropriate and is the work technically sound?

Partly

Are the conclusions drawn adequately supported by the results?

Yes

Are sufficient details of methods and analysis provided to allow replication by others?

Partly

Reviewer Expertise:

Epidemiology, gametocyte biology

I confirm that I have read this submission and believe that I have an appropriate level of expertise to confirm that it is of an acceptable scientific standard, however I have significant reservations, as outlined above.

# Peer review: Reviewer response for version 2

**DOI:** 10.21956/wellcomeopenres.18379.r43475

**Published:** 2021-04-23

**Authors:** Linda E Amoah

**Affiliations:** 1 Immunology Department, Noguchi Memorial Institute for Medical Research, University of Ghana, Accra, Ghana

The responses to my first set of questions have led to a new set of questions/comments and suggestions.

 Based on this quotation: 'The total number of samples used in assessing the relationship between gametocyte density, MOI and mosquito infection prevalence was 37.' I think it is inappropriate to use all the samples used in the original study in this paper. This paper should focus only on the total number that gave the 4 ml of venous blood or the gametocyte positive individuals. It is very misleading to report all the numbers when only a fraction were/are the focus of this report, so then you can only show a map of where the few samples used in this study were obtained from.

 The discussion pertains mostly to the 84, I think this should reduce to the 37 whole blood donors. I also think that the title can remain if the prevalence of gametocytes is reported for the 37 individuals as opposed to the 2459 individuals.

Is the work clearly and accurately presented and does it cite the current literature?

Partly

If applicable, is the statistical analysis and its interpretation appropriate?

Partly

Are all the source data underlying the results available to ensure full reproducibility?

Yes

Is the study design appropriate and is the work technically sound?

Partly

Are the conclusions drawn adequately supported by the results?

Partly

Are sufficient details of methods and analysis provided to allow replication by others?

Partly

Reviewer Expertise:

molecular biology, malaria transmission, diagnostics and vaccine design

I confirm that I have read this submission and believe that I have an appropriate level of expertise to confirm that it is of an acceptable scientific standard, however I have significant reservations, as outlined above.

# Peer review: Reviewer response for version 2

**DOI:** 10.21956/wellcomeopenres.18379.r43472

**Published:** 2021-04-23

**Authors:** Lynn Grignard

**Affiliations:** 1 Department of Infection Biology, Faculty of Infectious and Tropical Diseases, London School of Hygiene and Tropical Medicine, London, UK

I have no further comments, thank you for incorporating reviewers' comments.

Is the work clearly and accurately presented and does it cite the current literature?

Yes

If applicable, is the statistical analysis and its interpretation appropriate?

Yes

Are all the source data underlying the results available to ensure full reproducibility?

Partly

Is the study design appropriate and is the work technically sound?

Yes

Are the conclusions drawn adequately supported by the results?

Yes

Are sufficient details of methods and analysis provided to allow replication by others?

Yes

Reviewer Expertise:

Malaria, molecular biology, transmission biology

I confirm that I have read this submission and believe that I have an appropriate level of expertise to confirm that it is of an acceptable scientific standard.

# Peer review: Reviewer response for version 1

**DOI:** 10.21956/wellcomeopenres.17910.r42718

**Published:** 2021-04-06

**Authors:** Lisa Ranford Cartwright

**Affiliations:** 1 Institute of Biodiversity, Animal Health and Comparative Medicine, College of Medical, Veterinary and Life Sciences, University of Glasgow, Glasgow, UK

**Summary:**

 The research aims were to investigate the prevalence of P. falciparum gametocyte carriage in asymptomatic schoolchildren in a region of Kenya and to investigate any association between the gametocyte density, the genetic complexity (of the total parasite population present in an individual) and their ability to infect mosquitoes following membrane feeding. The main findings of the paper are in agreement with previously published research, in that younger children had a higher likelihood of gametocyte carriage, that male children had a higher likelihood of being parasite positive than females, and that mosquito infection was positively correlated with gametocyte density. One finding of a positive association between genetic complexity of infection and mosquito infection prevalence was not in agreement with previous research.

**Study design:**

 Asymptomatic individuals were identified by a screening of almost 5000 school children using an RDT, followed by microscopy to identify those with gametocytes present.  The choice of RDT to screen for asymptomatic infections is problematic because such tests have a higher threshold for parasite detection - the specific RDT chosen seems to reliably detect parasite densities above 200 parasites per microlitre, but has a lower sensitivity below that (Djalle et al 2014 BMC Inf. Dis.). This makes it likely that individuals with lower level infections will be missed. The identification of gametocyte carriers using microscopy has also low sensitivity. The end result of the selection of these two methods is a likely underestimation of asymptomatic carriage of parasites and of gametocytes; only those individuals with higher asexual and gametocyte density will be included in the study. That risks bias in the analysis and conclusions since many asymptomatically-infected individuals would not be sampled. This issue needs to be discussed.

**Sample size:**

 Perhaps as a result of the screening, which would miss lower level infections, there is an extremely small sample size. Of the 84 individuals carrying microscopically-detectable gametocytes, only 37 were used for mosquito infections. Were these 37 selected for the mosquito infection study based on any factor, or was it random?  For example, did the 37 have the same age and gender split as the total gametocyte positive group? Of those 37, MOI data were only available for 22 individuals. The majority of the statistical analysis is therefore done on 22 data points only.

**Data inconsistency:**

 There are some errors or inconsistencies in the data presented. The main one is in the MOI numbers that have been used for the analysis. I applaud the inclusion of all the raw data. However the calculations of MOI in the supplementary data (allele counts) do not match those used/ presented in the main paper in 11 of the 23 individuals e.g. Donor#3 has one allele listed for microsatellite TA60, but the MOI for that locus is given as 2. For unexplained reasons, the allele sizes for some microsatellites have been duplicated (so two alleles of the same size are listed and counted). Very high MOI values are not supported by the raw data given - for example, donor 3 has an MOI of 12 but the maximum number of alleles at any one locus is 10).

**Incomplete data:**

 Only the percentage of mosquitoes infected is given, which does not give any idea about how robust the differences are. The numbers infected/ dissected should be given, and statistical analysis would be better performed based on numbers rather than percentage infections, which would reflect the likely accuracy of the figures used.

**Statistical analyses:**

 The statistical analyses are not well explained and could be improved. It is unclear why age and gender have been examined separately in the prevalence of infection and gametocyte carriage data. Although unlikely,  there could be an imbalance of genders in the two age groups that would bias the results.

 It is difficult to understand exactly how the infection data and correlations with MOI and gametocyte density have been carried out, and thus how robust the conclusions are given the very small sample size. For example, were the variables of gender and age also included in the regression analyses? The plot (Figure 3) is not convincing in supporting the conclusion that MOI is linked to infection prevalence (R ^2^ value of 0.2032), and the gametocyte density vs infection also has a low R ^2^ value, with the plot indicating the positive correlation is mainly due to three individuals with >30% infected mosquitoes, high gametocyte density and high MOI.  The regression outputs show a somewhat surprising) significant association of gametocyte density and MOI on infection prevalence. However, it is likely that incorrect MOI values have been used. More information is needed on the modelling method used (linear regression? GLM?) and the factors included. The statistical analysis needs to be clarified and repeated using corrected data.

**Overall:**

 The findings of the paper reinforce what has been previously reported from previous studies.

**Minor points for clarification/correction:**

1. Was each child only included once in the data set ie the same child was not sampled in both years?
2. The material used for positive controls/ to generate standard curves for the CSP ELISA is not sufficiently described. In addition, what is the threshold of this test for detection of parasites at the oocyst stage of infection?
3. The total number of female children examined/ positive is given in the text as 657/2457 but the total number of females examined is 2459 in the table.

Is the work clearly and accurately presented and does it cite the current literature?

Partly

If applicable, is the statistical analysis and its interpretation appropriate?

Partly

Are all the source data underlying the results available to ensure full reproducibility?

Yes

Is the study design appropriate and is the work technically sound?

Partly

Are the conclusions drawn adequately supported by the results?

Partly

Are sufficient details of methods and analysis provided to allow replication by others?

No

Reviewer Expertise:

Genetics and transmission biology of P. falciparum.

I confirm that I have read this submission and believe that I have an appropriate level of expertise to state that I do not consider it to be of an acceptable scientific standard, for reasons outlined above.

# Peer review: Reviewer response for version 1

**DOI:** 10.21956/wellcomeopenres.17910.r42272

**Published:** 2021-02-17

**Authors:** Teun Bousema

**Affiliations:** 1 Department of Medical Microbiology, Radboud University Nijmegen Medical Center, Nijmegen, The Netherlands

The authors present an analysis on infectivity from a limited number of gametocyte carriers from a narrow age range. Gametocyte quantification is done by microscopy, not ideal for this, but nevertheless very strongly associated with transmission risk. The data collection was, as indicated, for other studies. Nevertheless, the authors present one interesting finding. The article could be shortened considerably and can improve the presentation of the main finding of interest, the association between MOI and infectivity to mosquitoes. I have a large number of specific comments. 

 The authors report a higher parasite prevalence in males. This, if based on a good random selection of the population, is of interest and in line with recent findings from Uganda (Briggs eLIFE 2020). Any risk of bias in the source population should be described. What proportion of invited participants participated and is there any (self-) bias possible?

 Prevalence of gametocytes can be presented for the entire population or from malaria-infected individuals. Both are of relevance, the former to estimate the age-structure in the ‘infectious reservoir’ and the latter highlighting differences in parasite density, gametocyte production or immunity. Both estimates should thus be presented. However, asexual parasite prevalence among parasite positive individuals is less interesting. All gametocyte carriers are asexual parasite carriers or very recently were.

**Abstract:**

- ‘challenging An. gambiae s.l.’ is an unusual choice of words. Allowing to feed or offering blood to… is more conventional.
- The abstract would also benefit from a bit more detail on the predictors of mosquito infection rates. Now only the statistically significat predictors are mentioned but some narrative would help the readers.
- Also the (modest) sample size should be mentioned in the abstract. Both in terms of experiments and numbers of mosquitoes dissected.

**Introduction:**

- ‘As a result, this category is most…’ I assume age group is meant. This could be clarified.
- The phrasing ‘Some studies have reported a positive association between mosquito infection rates of *P. falciparum* and gametocyte density, particularly at high gametocyte concentrations. However, at low gametocyte concentrations, a varying and less strong association is reported.’ Is a bit misleading. As is quite clearly described in the literature (Bradley *et al.* eLIFE 2018 and Johnston PLoS Comp Biol 2012) ^1^^,^^2^, there is a clear positive association with sporadic infections at gametocyte densities below 10 gametocytes/uL.
- ‘Two common characteristics of asymptomatic malaria infections in endemic settings are the prevalence of varying levels of gametocyte carriage among different age categories due to anti-parasite immunity and high rates of polyclonal infections’ is a complicated sentence best broken up in 2 (or even 3).
- ‘In order to ultimately eliminate malaria, interventions geared towards interrupting the disease transmission through efficient and effective identification and treatment of both asymptomatic and symptomatic parasite carriers will be of immense importance’ is over-stating the evidence. Countries have eliminated malaria without a specific focus on asymptomatic infections. One can indeed expect that elimination would be accelerated by also targeting asymptomatic infections. I would propose parasite transmission rather than disease transmission, the symptoms are not transmitted by mosquitoes.

**Methods:**

- Figure 1, the study map, is interesting if all schools had a decent sample size. The number of observations (median, range) should be presented in the legend.
- ‘This sample size was obtained based on the number of study participants within the designated study area that consented to partake in the study.’ This is not very meaningful. How was the sample size decided upon? Did the authors aim to reach a certain sample size to address the current study questions or was the sample size decided upon to support other study questions?
- In the methods, please indicate what anticoagulant was used for phlebotomy and indicate the number of microscopy fields screened for gametocytes (or the number of white blood cells counted against) to give an idea of assay sensitivity.
- In the methods, please indicate the source of water-jacketed feeders and capacity (volume).
- It would be nice to understand why a qualitative assay (ELISA) was used as read-out and whether semi-quantitative read-out, as in Graumans MalJ 2017 was considered. ^3^
- Please indicate what positive controls were used for the ELISA (source).
- Some details on the (minimum) number of dissected mosquitoes would be expected in the results section. In general, just present % infectious and % infected mosquitoes with more reference to denominators. The findings probably hold their value but at the moment it is unknown what feeding performance was and how mosquito survivorship may have affected the precision of outcomes.
- Any age-patterns, concluded to be non-exist here, are likely to be obscured by the small age range examined.
- It is unclear to me how mixed infections were examined. Is this by PCR or by microscopy?
- All references to gametocyte carriage should, at least early in the results and discussion section, be referred to as ‘microscopy gametocyte prevalence’ to make sure the reader understands the limitations of the diagnostic used.
- Comparisons of gametocyte prevalence by sex could be presented adjusted and unadjusted by total parasite density.
- Table 1 should be simplified. ‘Positive’ and ‘negative’ in the first rows are confusing and probably mutually exclusive.
- The presentation of asexual parasite prevalence and gametocyte prevalence (e.g. 99.99% vs 8% is not informative. Just present asexual prevalence and gametocyte prevalence by gender and age group.
- I would present for age and sex separately (so girls 5-9 vs boys 5-9 and girls 10-15 vs boys 10-15) even if only gender is statistically significantly associated with risk.
- Figure 2 can be omitted. It is not relevant to the current story.
- Table 2 can be omitted and captured in the text.
- Table 3 is confusing. It suggests that gametocyte density is the reference for gametocyte density. I understand that one cannot calculate a correlation coefficient here but that is not the same as calling it a reference category.
- Figure 3 suggests that MOI is a factor independent of gametocytes and is directly associated with infectivity. MOI can either be associated with higher gametocyte density (which appears not to be the case) or higher infectivity for a given gametocyte density. The latter would be better displayed if categories of gametocyte densities (e.g. <10, 10-20 and 20+, broadly tertiles) are defined as well as categories of MOI and for each gametocyte, class infectivity is given for low, intermediate and high MOI. That would allow an interpretation if, for each gametocyte class, MOI is associated with higher infectivity.
- Table 4 requires information about scale. Is gametocyte density included per uL with or without log10 adjustment? Is MOI included as a continuous variable? That would not be entirely intuitive and I would rather see MOI in categories since there is no reason I know of to assume that the difference between an MOI of 1 and 2 (single clone versus multiclone and thus potentially competition between clones) is the same as that between an MOI of 10 and 11.

**Discussion:**

- The statement ‘Among the P. falciparum malaria positive individuals, males tended to be slightly overrepresented as both asexual 53.76% (764/1421) and gametocyte carriers 61.9% (52/84) as compared to females [asexual carriage; 46.23% (657/1421), gametocyte carriage; 38.1% (32/84)] is very confusing and should come with an estimate of statistical significance. There is no reason to add up asexuals and gametocytes to classify someone as parasite positive and then determine the proportion of these positives that is asexual positive. It just doesn’t make much sense biologically or epidemiologically.
- The part on gametocyte sex ratio can be removed from the discussion. It is interesting but has no relevance to the current study that didn’t assess sex ratio.
- The authors suggest competition between virulent and less virulent (defined as transmissible) strains as a mechanism underlying the association between MOI and mosquito infection rates. They cite references 49-51 but none of these, as far as I know, prove this association. They merely hint at inter-strain competition that is most likely to occur through increased investment in gametocytes or gametocytes of a certain sex.

Is the work clearly and accurately presented and does it cite the current literature?

Yes

If applicable, is the statistical analysis and its interpretation appropriate?

Partly

Are all the source data underlying the results available to ensure full reproducibility?

Yes

Is the study design appropriate and is the work technically sound?

Partly

Are the conclusions drawn adequately supported by the results?

Yes

Are sufficient details of methods and analysis provided to allow replication by others?

Partly

Reviewer Expertise:

Epidemiology, gametocyte biology

I confirm that I have read this submission and believe that I have an appropriate level of expertise to confirm that it is of an acceptable scientific standard, however I have significant reservations, as outlined above.

## References

[1] : Predicting the likelihood and intensity of mosquito infection from sex specific Plasmodium falciparum gametocyte density. *eLife*.2018;7: 10.7554/eLife.34463 10.7554/eLife.34463 PMC601325529848446

[2] : Malaria's missing number: calculating the human component of R0 by a within-host mechanistic model of Plasmodium falciparum infection and transmission. *PLoS Comput Biol*.2013;9(4) : 10.1371/journal.pcbi.1003025 e1003025 10.1371/journal.pcbi.1003025 23637586PMC3630126

[3] : Semi-high-throughput detection of Plasmodium falciparum and Plasmodium vivax oocysts in mosquitoes using bead-beating followed by circumsporozoite ELISA and quantitative PCR. *Malaria Journal*.2017;16(1) : 10.1186/s12936-017-2011-9 10.1186/s12936-017-2011-9 28877707PMC5588624

# Response

**Published:** 2021-03-18

**Authors:** Jeremy Herren

The authors present an analysis on infectivity from a limited number of gametocyte carriers from a narrow age range. Gametocyte quantification is done by microscopy, not ideal for this, but nevertheless very strongly associated with transmission risk. The data collection was, as indicated, for other studies. Nevertheless, the authors present one interesting finding. The article could be shortened considerably and can improve the presentation of the main finding of interest, the association between MOI and infectivity to mosquitoes. I have a large number of specific comments. 

*Many thanks for these comments, we have done our best to address the issues raised.*

 The authors report a higher parasite prevalence in males. This, if based on a good random selection of the population, is of interest and in line with recent findings from Uganda (Briggs eLIFE 2020). Any risk of bias in the source population should be described. What proportion of invited participants participated and is there any (self-) bias possible?

*Unfortunately, we did not keep information on the proportion of invited participants that participated. The overwhelming majority of invited participants participated in the RDT test. Upon reflection and discussion we are not able to find anything that would have led to bias.*

 Prevalence of gametocytes can be presented for the entire population or from malaria-infected individuals. Both are of relevance, the former to estimate the age-structure in the ‘infectious reservoir’ and the latter highlighting differences in parasite density, gametocyte production or immunity. Both estimates should thus be presented. However, asexual parasite prevalence among parasite positive individuals is less interesting. All gametocyte carriers are asexual parasite carriers or very recently were.

**Abstract:**

- ‘challenging An. gambiae s.l.’ is an unusual choice of words. Allowing to feed or offering blood to… is more conventional.

*We have modified this to be, “After offering gametocyte positive blood to An. gambiae s.l. by membrane feeding assay, our results indicated that 68.1% of the variation in mosquito infection prevalence was accounted for by gametocyte density and MOI (R-SQR. = 0.681, p < 0.001).”*


* *

- The abstract would also benefit from a bit more detail on the predictors of mosquito infection rates. Now only the statistically significat predictors are mentioned but some narrative would help the readers.

*We have revised as follows, “Gametocyte presence in the host peripheral blood is a significant factor of malaria parasite transmission. However, this does not translate to infectivity in the mosquito vector. Some of the predictors of mosquito infectivity include, the gametocyte sex-ratio and density, multiplicity of infection (MOI), and host and vector anti-parasite immunity”*

  

- Also the (modest) sample size should be mentioned in the abstract. Both in terms of experiments and numbers of mosquitoes dissected.

*We have revised as follows, “37 microscopy positive gametocyte carriers were selected to feed laboratory reared An. gambiae s.l. mosquitoes using membrane feeding method. 3395 fully fed mosquitoes were used to do ELISA for determining mosquito infection prevalence.”*

**Introduction:**

- ‘As a result, this category is most…’ I assume age group is meant. This could be clarified.
- The phrasing ‘Some studies have reported a positive association between mosquito infection rates of *P. falciparum* and gametocyte density, particularly at high gametocyte concentrations. However, at low gametocyte concentrations, a varying and less strong association is reported.’ Is a bit misleading. As is quite clearly described in the literature (Bradley *et al.* eLIFE 2018 and Johnston PLoS Comp Biol 2012) ^1^ ^,^ ^2^, there is a clear positive association with sporadic infections at gametocyte densities below 10 gametocytes/uL.

*This has been revised and the stated references are added in the text.*


* *

- ‘Two common characteristics of asymptomatic malaria infections in endemic settings are the prevalence of varying levels of gametocyte carriage among different age categories due to anti-parasite immunity and high rates of polyclonal infections’ is a complicated sentence best broken up in 2 (or even 3).

*We have revised as follows, “In malaria endemic settings, asymptomatic infections characterized by high rates of polyclonal infections and variations in gametocyte carriage among different age categories is not uncommon [15, 28, 29]. Variations in gametocyte densities among the different age categories can be partly explained by the age-related anti-parasite immunity due to repeated exposure among the elderly children and adults age groups [21].* *”*

  

- ‘In order to ultimately eliminate malaria, interventions geared towards interrupting the disease transmission through efficient and effective identification and treatment of both asymptomatic and symptomatic parasite carriers will be of immense importance’ is over-stating the evidence. Countries have eliminated malaria without a specific focus on asymptomatic infections. One can indeed expect that elimination would be accelerated by also targeting asymptomatic infections. I would propose parasite transmission rather than disease transmission, the symptoms are not transmitted by mosquitoes.

*This section is revised in the introduction section in accordance with the comment above.*

**Methods:**

- Figure 1, the study map, is interesting if all schools had a decent sample size. The number of observations (median, range) should be presented in the legend.

*This has been added to the map *

- ‘This sample size was obtained based on the number of study participants within the designated study area that consented to partake in the study.’ This is not very meaningful. How was the sample size decided upon? Did the authors aim to reach a certain sample size to address the current study questions or was the sample size decided upon to support other study questions?

*The samples included in this study were determined to answer other study questions. So this current study is a sub study that was concurrently undertaken using the same samples.*


* *

- In the methods, please indicate what anticoagulant was used for phlebotomy and indicate the number of microscopy fields screened for gametocytes (or the number of white blood cells counted against) to give an idea of assay sensitivity.

*Heparin was used as anticoagulant, gametocytes were counted against 1000 white blood cells and the counts converted to parasites/μ* *L* *assuming a density of 8000 WBCs/μ* *L.*

  

- In the methods, please indicate the source of water-jacketed feeders and capacity (volume).

*Added to methods.*

- It would be nice to understand why a qualitative assay (ELISA) was used as read-out and whether semi-quantitative read-out, as in Graumans MalJ 2017 was considered. ^3^

*We were not convinced that the ELISA test gave consistently accurate semi-quantitative read-outs on tests carried out with positive control dilutions.*

- Please indicate what positive controls were used for the ELISA (source).

*Pf-PC was used as a positive control for ELISA (BEI resources).*

  

- Some details on the (minimum) number of dissected mosquitoes would be expected in the results section. In general, just present % infectious and % infected mosquitoes with more reference to denominators. The findings probably hold their value but at the moment it is unknown what feeding performance was and how mosquito survivorship may have affected the precision of outcomes.

*We have amended the results based on this comment.*

- Any age-patterns, concluded to be non-exist here, are likely to be obscured by the small age range examined.

*We have added this in the discussion section.*


* *

- It is unclear to me how mixed infections were examined. Is this by PCR or by microscopy?

*Mixed infections were detected using RDT test and microscopy examination* *.*

  

- All references to gametocyte carriage should, at least early in the results and discussion section, be referred to as ‘microscopy gametocyte prevalence’ to make sure the reader understands the limitations of the diagnostic used.

*We have addressed this by adding microscopy gametocyte prevalence for the purpose of clarity* *.*


*  *

- Comparisons of gametocyte prevalence by sex could be presented adjusted and unadjusted by total parasite density.
* *

*They have now also been presented as unadjusted.*

- Table 1 should be simplified. ‘Positive’ and ‘negative’ in the first rows are confusing and probably mutually exclusive.

*These has been separated from the rest of the table.*

  

- The presentation of asexual parasite prevalence and gametocyte prevalence (e.g. 99.99% vs 8% is not informative. Just present asexual prevalence and gametocyte prevalence by gender and age group.

*All the presentations have been revised.*

  

- I would present for age and sex separately (so girls 5-9 vs boys 5-9 and girls 10-15 vs boys 10-15) even if only gender is statistically significantly associated with risk.

*We have revised as follows, “* *However, considering the age versus sex distribution of gametocyte carriage, no significant difference was found by comparing the male (5-9 years) [53.65% (382/712)] to female (5-9 years) [46.35% (330/712)] and male (10-15 years) [53.88% (382/709)] to female (10-15 years) [46.12% (327/709)] (p-value = 0.958).”*

- Figure 2 can be omitted. It is not relevant to the current story.
* *
*We did not omit this because Reviewer_1 was interested to see the infection pattern and recommended we add some details about figure two. This is the reason I did not omit it.*

 

- Table 2 can be omitted and captured in the text.

*Table 2 is omitted and captured in the text and the discussion section as well.*

  

- Table 3 is confusing. It suggests that gametocyte density is the reference for gametocyte density. I understand that one cannot calculate a correlation coefficient here but that is not the same as calling it a reference category.

*‘’Ref’’ were removed that was an oversight.*


* *

- Figure 3 suggests that MOI is a factor independent of gametocytes and is directly associated with infectivity. MOI can either be associated with higher gametocyte density (which appears not to be the case) or higher infectivity for a given gametocyte density. The latter would be better displayed if categories of gametocyte densities (e.g. <10, 10-20 and 20+, broadly tertiles) are defined as well as categories of MOI and for each gametocyte, class infectivity is given for low, intermediate and high MOI. That would allow an interpretation if, for each gametocyte class, MOI is associated with higher infectivity.

*This was considered during the data analysis. However, we could not present the data as you have suggested due to the very small number of samples analyzed in this study. However, this will be considered in the subsequent studies.*

  

- Table 4 requires information about scale. Is gametocyte density included per uL with or without log10 adjustment? Is MOI included as a continuous variable? That would not be entirely intuitive and I would rather see MOI in categories since there is no reason I know of to assume that the difference between an MOI of 1 and 2 (single clone versus multiclone and thus potentially

competition between clones) is the same as that between an MOI of 10 and 11.

*This is an important point and will provide detailed information. However, considering the low sample size in this study we could not present the MOI as a category.*

**

**Discussion:**

- The statement ‘Among the P. falciparum malaria positive individuals, males tended to be slightly overrepresented as both asexual 53.76% (764/1421) and gametocyte carriers 61.9% (52/84) as compared to females [asexual carriage; 46.23% (657/1421), gametocyte carriage; 38.1% (32/84)] is very confusing and should come with an estimate of statistical significance. There is no reason to add up asexuals and gametocytes to classify someone as parasite positive and then determine the proportion of these positives that is asexual positive. It just doesn’t make much sense biologically or epidemiologically.

*We have revised as follows, “* *Among the P. falciparum gametocyte positive individuals, males tended to be slightly overrepresented 61.9% (52/84) as compared to females 38.1% (32/84). However, this is not statistically significant.”*


* *

- The part on gametocyte sex ratio can be removed from the discussion. It is interesting but has no relevance to the current study that didn’t assess sex ratio.

*This part is revised and removed most of the content *

- The authors suggest competition between virulent and less virulent (defined as transmissible) strains as a mechanism underlying the association between MOI and mosquito infection rates. They cite references 49-51 but none of these, as far as I know, prove this association. They merely hint at inter-strain competition that is most likely to occur through increased investment in gametocytes or gametocytes of a certain sex.

*We have now indicated that this may be linked to intense inter-strain competition due to increased investment in gametocytes and multiple clone infections (MOI) that could favour emergence of highly transmissible or virulent parasite strains thereby increase mosquito infectivity [48, 49, 50].*

# Peer review: Reviewer response for version 1

**DOI:** 10.21956/wellcomeopenres.17910.r42224

**Published:** 2021-02-01

**Authors:** Lynn Grignard

**Affiliations:** 1 Department of Infection Biology, Faculty of Infectious and Tropical Diseases, London School of Hygiene and Tropical Medicine, London, UK

The authors present a well written manuscript titled "Prevalence of asymptomatic *P. falciparum *gametocyte

 carriage in schoolchildren and assessment of the association between gametocyte density, multiplicity of infection and mosquito infection prevalence". The authors clearly define and outline their research question and background and give in depth details of the methods used in the study. The methods are described in great length, but there is the occasional omission of some detail. For example, in the section describing the membrane feeding assay, the authors should cite publications they have based their assay on (the protocol has not been newly developed by the authors in this publication) and state their modifications. Small details like the type of anticoagulant (heparin!) in the blood collection tubes is important for researchers to be able to replicate the experiment. The following section on ELISA contains too many details and could be streamlined i.e. you say you incubate "overnight" and then in the next sentence "the next morning"  - as you mention the overnight incubation, there is no need to add "the next morning", you tell the reader that you equilibrate your samples to RT - and detail each step as you would in a lab protocol or SOP. These are just a couple of examples and the whole section needs shortening. 

 The results section is well structured and the data is adequately interpreted. I seem to be unable to find asexual parasite densities - overall but also for the subgroups. 

 Could the authors please explain/give more detail about the samples: There were 84 gametocyte carriers - how many infected mosquitoes? There is feeding data for 37 individuals (is that because only 37/84 infected mosquitoes?). Of these 37 infectious individuals, only 22 have MOI data (again why is that?). Also, gametocyte densities might be better in median and IQR or geometric mean and 95% CI. Equally, MOI is more informative as a median and IQR compared to mean.

 In the discussion, the authors say that "High infection prevalence was observed among mosquitoes that fed on carriers with high gametocyte densities." - this is true but looking at their data apart from 2/37 individuals all gametocyte carriers were below 200 gam/ul - and of the ones above 100 gam/ul only 4 infected more than the mean mosquito infection rate. 

 The manuscript could be improved as follows: The discussion needs a section on limitations. These should include and discuss (not exclusively): the use of microscopy to identify gametocyte carriers, the small number of MOI data points, the relatively small number of data points/category, membrane feeding versus skin feeding, MOI of asexuals vs gametocytes, relatively high MOI in light of transmission area.

Is the work clearly and accurately presented and does it cite the current literature?

Yes

If applicable, is the statistical analysis and its interpretation appropriate?

Yes

Are all the source data underlying the results available to ensure full reproducibility?

Partly

Is the study design appropriate and is the work technically sound?

Yes

Are the conclusions drawn adequately supported by the results?

Yes

Are sufficient details of methods and analysis provided to allow replication by others?

Yes

Reviewer Expertise:

Malaria, molecular biology, transmission biology

I confirm that I have read this submission and believe that I have an appropriate level of expertise to confirm that it is of an acceptable scientific standard, however I have significant reservations, as outlined above.

# Response

**Published:** 2021-03-18

**Authors:** Jeremy Herren

The authors present a well written manuscript titled "Prevalence of asymptomatic *P. falciparum *gametocyte

 carriage in schoolchildren and assessment of the association between gametocyte density, multiplicity of infection and mosquito infection prevalence". The authors clearly define and outline their research question and background and give in depth details of the methods used in the study. The methods are described in great length, but there is the occasional omission of some detail. For example, in the section describing the membrane feeding assay, the authors should cite publications they have based their assay on (the protocol has not been newly developed by the authors in this publication) and state their modifications.

 Small details like the type of anticoagulant (heparin!) in the blood collection tubes is important for researchers to be able to replicate the experiment.

 The following section on ELISA contains too many details and could be streamlined i.e. you say you incubate "overnight" and then in the next sentence "the next morning"  - as you mention the overnight incubation, there is no need to add "the next morning", you tell the reader that you equilibrate your samples to RT - and detail each step as you would in a lab protocol or SOP. These are just a couple of examples and the whole section needs shortening. 

 The results section is well structured and the data is adequately interpreted. I seem to be unable to find asexual parasite densities - overall but also for the subgroups. 

*The asexual parasite densities were not included here because the study was centered on trying to find the relationship between gametocyte density, MOI and infectivity. So, we tend to only screen the potential asymptomatic carriers then if positive, we screen for gametocyte carriage and those that were positive for gametocyte carriage, the density of the gametocytes in the blood was determined.*

 Could the authors please explain/give more detail about the samples: There were 84 gametocyte carriers - how many infected mosquitoes?

*This was stated in the method section. Each sample (37 in total) were fed to 100 mosquitoes. After feeding, the fully fed mosquitoes were kept for about 8 to 10 days. The number of mosquitoes infected was 463 mosquitoes.*

 There is feeding data for 37 individuals (is that because only 37/84 infected mosquitoes?).

*This is because only 37 individuals consented to providing extra venous blood used during mosquito feeding and the same samples were used to extract genomic DNA for the microsatellite genotyping analysis.*

 Of these 37 infectious individuals, only 22 have MOI data (again why is that?).

*Only those samples used for the mosquito feeding were used to extract genomic DNA for the microsatellite genotyping analysis. However, the 15 samples failed to amplify due to power failure and degradation of the stored DNA samples. Therefore, the MOI data was obtained for only 22 samples.*

 Also, gametocyte densities might be better in median and IQR or geometric mean and 95% CI. Equally, MOI is more informative as a median and IQR compared to mean.

*We have included this in the results sections*

 In the discussion, the authors say that "High infection prevalence was observed among mosquitoes that fed on carriers with high gametocyte densities." - this is true but looking at their data apart from 2/37 individuals all gametocyte carriers were below 200 gam/ul - and of the ones above 100 gam/ul only 4 infected more than the mean mosquito infection rate. 

*This statement was revised to match the stated comment. “Infection prevalence tends to be relatively higher among mosquitoes that fed on carriers with high gametocyte densities (> 20 Gam/uL.”*

 The manuscript could be improved as follows:

 The discussion needs a section on limitations. These should include and discuss (not exclusively):

 the use of microscopy to identify gametocyte carriers,

 the small number of MOI data points, the relatively small number of data points/category,

 membrane feeding versus skin feeding,

 MOI of asexuals vs gametocytes, relatively high MOI in light of transmission area.

*We have tried to address these points by including the section on limitations covering all the major points stated above.*

# Peer review: Reviewer response for version 1

**DOI:** 10.21956/wellcomeopenres.17910.r41941

**Published:** 2021-01-25

**Authors:** Linda E Amoah

**Affiliations:** 1 Immunology Department, Noguchi Memorial Institute for Medical Research, University of Ghana, Accra, Ghana

**Title:**

- The title must include 'Kenya' where the study was conducted.

**Abstract:**

- 'microscopy gametocyte' What does this mean?
- Some results are discussed as past tense and others as present. Consistency is required.
- 'MOI statistically, the 'statistically' can be removed.

**Introduction:**

- 'genetically diverse multiple' Should be multiple genetically diverse.
- The paragraph beginning 'it has also been.....' should be rephrased.

**Methods:**

- the study site description is very scanty.
- Was this a longitudinal study or cross sectional study?
- How many schools were used in the study?

Study subjects and sample collection:

- This section should be elaborated to include the number of samples collected for each aspect of the study.
- What exactly is KM? Is it Km?
- The paragraph beginning 'Blood samples... ' should be rephrased and include the volume of blood drawn.
- How much blood was spotted on the filter paper?
- No molecular determination of gametocyte prevalence was made. Is it a known fact that gametocyte prevalence and densities are not adequately captured by microscopy?

Experimental infection of mosquitoes:

- The description is very difficult to understand it should be rewritten to enhance clarity.
- The section begins with the statement 4 ml blood collected from ALL microscopy positive gametocyte carriers were immediately fed to mosquitoes (this number is 84 from the previous section). Then towards the middle, there is a statement that only 37 samples were fed to mosquitoes.
- Why were only 37 samples fed to mosquitoes?
- Then if female mosquitoes are used for the feeding experiment, why are male insectary reared mosquitoes used as negative controls?
- Concentration should not be written as volume 'a concentration of 40 ul.....'.
- Were individual mosquitoes tested in the ELISA? This should be clearly written,
- I am not sure how strong an analysis of 37 samples divided into subgroups would be.

Microsatellite genotyping:

- This section is poorly written. There is no consistency in reporting SI units. There are several instances where SI units are written directly after the number, without a space.
- Then there is a confusing statement: 'the samples analyzed here are part of those used in our previous study'? are these archived samples. are all the 4881 samples archived? or collected for this study?
- Why were only 37 samples analyzed?
- If 15 samples failed to amplify, that would leave only 22 samples.
- Is it possible to divide 22 into multiple groups and obtain statistically significant results?
- The map should be properly labelled so that all sublocations where samples were collected are labelled/named. 
- There is no primary school located in 'C'.

**Results:**

- It would be very informative to separate the *P. malariae *infections from the *P. ovale *infections.
- Comparisons are being made but it seems the seasons were not considered in the analysis.
- The study spanned over 2 years and across a number of peak and off peak seasons and across communities with varying malaria parasite prevalence. The analysis should have taken all these into consideration.
- Figure 2 needs more information. % is indicated but a fraction should be either added as a foot note or supplementary file. this information is partially contained within the map but even with that, prevalence is grouped as a range. the exact prevalence per school per month should be provided. the supplementary table has the same % data as the figure.
- The major flaw in this study is the very small number of samples used for the assessment of the association between gametocyte density, MOI and infection prevalence.  22 samples are too small to use to identify significant associations and risk factors.

**Discussions:**

- It is difficult for me to comment on the discussion as I have major challenges with the results and the methods.

More details are required to support the interpretation of the supplementary tables. footnotes and description of what items ate in the columns and rows and what the numbers represent

Is the work clearly and accurately presented and does it cite the current literature?

Partly

If applicable, is the statistical analysis and its interpretation appropriate?

Partly

Are all the source data underlying the results available to ensure full reproducibility?

Yes

Is the study design appropriate and is the work technically sound?

Partly

Are the conclusions drawn adequately supported by the results?

Partly

Are sufficient details of methods and analysis provided to allow replication by others?

Partly

Reviewer Expertise:

molecular biology, malaria transmission, diagnostics and vaccine design

I confirm that I have read this submission and believe that I have an appropriate level of expertise to confirm that it is of an acceptable scientific standard, however I have significant reservations, as outlined above.

# Response

**Published:** 2021-03-18

**Authors:** Jeremy Herren

**Title:**

- The title must include 'Kenya' where the study was conducted.

*Title modified as follows:*

**Prevalence of asymptomatic *P. falciparum *gametocyte **carriage among schoolchildren in Mbita, Western Kenya and assessment of the association between gametocyte density, multiplicity of infection and mosquito infection prevalence.**

**Abstract:**

- 'microscopy gametocyte' What does this mean?

*We have opted to use gametocyte prevalence as determined by microscopy to avoid any confusion.*

  

- Some results are discussed as past tense and others as present. Consistency is required.

*This have been addressed for the purpose of consistency*

  

- 'MOI statistically, the 'statistically' can be removed.

*The sentence is rephrased as “MOI significantly”*

**Introduction:**

- 'genetically diverse multiple' Should be multiple genetically diverse.

*The sentence is rephrased as “genetically diverse”*

  

- The paragraph beginning 'it has also been.....' should be rephrased.

*Rephrased as, “Parasite sex ratio (ratio of male and female gametocyte density) was reported to influence the transmission potential of P. falciparum parasite”*

**Methods:**

- the study site description is very scanty.

*Further information about the study site like the main economic activities, and housing systems were added.*

  

- Was this a longitudinal study or cross sectional study?

*This was a cross-sectional study employing participants from the primary schools within the study area.*

  

- How many schools were used in the study?

*Study participants were recruited from 41 primary schools including public and private schools.*

 Study subjects and sample collection:

- This section should be elaborated to include the number of samples collected for each aspect of the study.

*We have included this information in each of the specific sections of the study for clarity.*

  

- What exactly is KM? Is it Km?

*Km for kilometer, we have now said “kilometer” to limit any confusion.*

  

- The paragraph beginning 'Blood samples... ' should be rephrased and include the volume of blood drawn.

*100 μL of blood samples were also collected on filter papers (two spots per paper)*

  

- How much blood was spotted on the filter paper?

*100 μL of blood samples were also collected on filter papers (two spots per paper) (Whatman 3 MM; Whatman, Maidstone, United Kingdom) for DNA extraction.*

  

- No molecular determination of gametocyte prevalence was made. Is it a known fact that gametocyte prevalence and densities are not adequately captured by microscopy?

*Yes, this is a known fact from previous studies comparing molecular methods and microscopy and is based on the relatively low sensitivity of microscopy.*

 Experimental infection of mosquitoes:

- The description is very difficult to understand it should be rewritten to enhance clarity.

*We have revised this section to ensure clarity but also using try to avoid missing out vital information for the purpose of reproducibility of the study.*

  

- The section begins with the statement 4 ml blood collected from ALL microscopy positive gametocyte carriers were immediately fed to mosquitoes (this number is 84 from the previous section). Then towards the middle, there is a statement that only 37 samples were fed to mosquitoes.

*The number of gametocyte positive individuals in the study population is 84. However, for the purpose of mosquito feeding, extra blood samples (4 mL) were collected only from those that have consented to that. Therefore, only 37 individuals have given consent to donate the extra 4 mL of venous blood to be used for the mosquito feeding hence the difference in the numbers.*

  

- Why were only 37 samples fed to mosquitoes?

*Those are the only individuals who consented to donate the 4 mL venous blood used for the mosquito feeding assay.*

  

- Then if female mosquitoes are used for the feeding experiment, why are male insectary reared mosquitoes used as negative controls?

*The male mosquitoes were used as negative controls because the male Anopheles mosquitoes do not feed on human blood and therefore are not infected with plasmodium parasite. Meaning they are clean and this a common practice and has been used in other studies.*

  

- Concentration should not be written as volume 'a concentration of 40 ul.....'.

*This was an error and has been corrected as “ The monoclonal antibody (MAb) peroxidase conjugate 27 (0.5 mg/mL Peroxidase Labelled Mouse Monoclonal Ab Pf2A10-CDC, CAT #: MRA-890, MR4/ATCC, Virginia, USA) was prepared in specified concentrations for Plasmodium falciparum at a volume of 40 μL in 10 mL blocking buffer for each plate.”*

  

- Were individual mosquitoes tested in the ELISA? This should be clearly written,

*Yes, and the information is added.*

  

- I am not sure how strong an analysis of 37 samples divided into subgroups would be.

*The sample size is relatively small due to some challenges. However, the statistical analysis employed in the study tends to have some level of strength though a more conclusive analysis can be carried out using larger sample sizes.*

 Microsatellite genotyping:

- This section is poorly written. There is no consistency in reporting SI units. There are several instances where SI units are written directly after the number, without a space.

*This has been corrected in the revised version.*

  

- Then there is a confusing statement: 'the samples analyzed here are part of those used in our previous study'? are these archived samples. are all the 4881 samples archived? or collected for this study?

*This statement is meant to show that we have published a first paper on genetic diversity and MOI in the study area before this one. That is what prompted this current publication and the samples used in the current study were all collected at the same time as those used in the previous publication. However, only the 37 samples used in the mosquito feeding were analyzed in this study.*

- Why were only 37 samples analyzed?

*This is because only 37 individuals consented to providing extra venous blood used during mosquito feeding and the same samples were used to extract genomic DNA for the microsatellite genotyping analysis.*

  

- If 15 samples failed to amplify, that would leave only 22 samples.

*Yes, and this was due to the degradation of those DNA samples.*

  

- Is it possible to divide 22 into multiple groups and obtain statistically significant results?

*From the analysis and even preliminary data analysis done prior to the final analysis, the statistical analysis was significant. The small sample sizes were also taken into strict consideration in selecting the test of significance methods used in the analysis.*

- The map should be properly labelled so that all sublocations where samples were collected are labelled/named. 

*We have not labelled the names of schools on the map since we do not have express permission to reveal their locations.*

- There is no primary school located in 'C'.

*There is a primary school however, no participant was recruited from that school because they did not fall within the inclusion criteria.*

**Results:**

- It would be very informative to separate the *P. malariae *infections from the *P. ovale *infections.

*Yes, we were aware of this. However, the RDT used in the mass screening only separates P. falciparum from the rest of the other species and only indicated a band for mixed infection of P. malariae and ovale as one. Since we are more interested about P. falciparum, we tend not to specifically screen for the other species.*

  

- Comparisons are being made but it seems the seasons were not considered in the analysis.

*While malaria is affected by seasons, this region is known to have high levels of infection year-round. Due to constraints regarding the number of samples, we believe it would not add much to the study to segregate samples by season.*

  

- The study spanned over 2 years and across a number of peak and off peak seasons and across communities with varying malaria parasite prevalence. The analysis should have taken all these into consideration.

*See above.*

- Figure 2 needs more information. % is indicated but a fraction should be either added as a foot note or supplementary file. this information is partially contained within the map but even with that, prevalence is grouped as a range. the exact prevalence per school per month should be provided. the supplementary table has the same % data as the figure.

*We have included this in the earlier version of the paper. However, based on other reviewers’ comments, we have only indicated the % on the figures. The data presented in the paper and all other raw data are available and can be shared once requested by any interested party.*

*The major flaw in this study is the very small number of samples used for the assessment of the association between gametocyte density, MOI and infection prevalence.  22 samples are too small to use to identify significant associations and risk factors.*

*The relatively small sample size used in this study was largely due to major constraints like obtaining consent from the study participants particularly for membrane feeding assay, sample transportation and storage and power supply problems leading to such low sample number. However, we have tried so hard that to use appropriate statistical methods taking into consideration the sample sizes to minimize any serious error. We have recommended a further multi-center study using a larger sample sizes to validate the findings from this study.*

**Discussions:**

- It is difficult for me to comment on the discussion as I have major challenges with the results and the methods.

More details are required to support the interpretation of the supplementary tables. footnotes and description of what items ate in the columns and rows and what the numbers represent

*We hope these issues have been address in the significant changes to the revised version.*
